# Replication of GWAS significant loci in a sub-Saharan African Cohort with early childhood caries: a pilot study

**DOI:** 10.1186/s12903-021-01623-y

**Published:** 2021-05-20

**Authors:** Olubukola O. Olatosi, Mary Li, Azeez A. Alade, Afolabi Oyapero, Tamara Busch, John Pape, Joy Olotu, Waheed Awotoye, Mohaned Hassan, Chinyere Adeleke, Wasiu L. Adeyemo, Elizabeth O. Sote, John R. Shaffer, Mary Marazita, Azeez Butali

**Affiliations:** 1 Department of Child Dental Health, Faculty of Dental Sciences, College of Medicine, University of Lagos, Lagos, Nigeria; 2 Department of Oral Pathology, Radiology and Medicine, College of Dentistry, University of Iowa, Iowa City , USA; 3 Iowa Institute of Oral Health Research, University of Iowa, Iowa City, IA USA; 4 Department of Epidemiology, College of Public Health, University of Iowa, Iowa City, IA USA; 5 Department of Preventive Dentistry, Faculty of Dentistry, Lagos State University College of Medicine, Lagos, Nigeria; 6 Department of Anatomy, University of Port Harcourt, Port Harcourt, Nigeria; 7 Department of Oral and Maxillofacial Surgery, University of Lagos, Lagos, Nigeria; 8 Department of Human Genetics, Graduate School of Public Health, University of Pittsburgh, Pittsburgh, PA USA; 9 Department of Oral and Craniofacial Sciences, School of Dental Medicine, University of Pittsburgh, Pittsburgh, PA USA

**Keywords:** Early childhood caries (ECC), Genetics, Prevalence, Risk factors, Severe early childhood caries

## Abstract

**Background:**

Early childhood caries (ECC) is a rapidly progressing form of dental infection and a significant public health problem, especially among socially and economically disadvantaged populations. This study aimed to assess the risk factors for ECC among a cohort of Sub-Saharan African children and to determine the role of genetics in the etiology of ECC.

**Methods:**

A sample of 691 children (338 with ECC, 353 without ECC, age<6years) was recruited from schools in Lagos, Nigeria. Socio-demographic, dental services utilization and infant dietary data were obtained with interviewer-administered questionnaire. Oral examination was conducted using the WHO oral health diagnostic criteria. Saliva samples were collected from the children for genetic analysis. Single nucleotide polymorphisms were selected from previous study for genotyping. Genetic association analyses to investigate the role of genetics in the etiology of ECC was done. Bivariate comparisons and Multivariate logistic regression analyses were conducted to assess associations between ECC and predictor variables, p<0.05.

**Results:**

Of the 338 children with ECC, 64 (18.9%) had Severe-Early Childhood Caries (S-ECC). Children aged 4859months comprised the highest proportion of subjects with ECC (165; 48.8%) and S-ECC (24; 37.5%) while female subjects had higher dt (3.132.56) and dmft values 3.272.64. ECC was significantly more prevalent among children who were breastfed at night12months (OR 3.30; CI 0.39, 4.75), those with no previous dental visit (OR 1.71; CI 0.24, 2.77),those who used sweetened pacifiers (OR 1.85; CI 0.91, 3.79) and those who daily consumed sugar-sweetened drinks/snacks (OR 1.35; CI0.09, 18.51). A suggestive increased risk for ECC (OR1.26, p=0. 0.0397) was observed for the genetic variant rs11239282 on chromosome 10. We also observed a suggestive reduced risk for ECC (OR0.80, p=0.03) for the rs131777 on chromosome 22. None of the genetic variants were significant after correction for multiple testing (Bonferroni p value p=0.004).

**Conclusions:**

Prolonged night-time breastfeeding, poor utilization of dental services and daily consumption of sugar were risk factors for ECC. Larger sample size is needed to confirm the results of the genetic analysis and to conduct genome wide studies in order to discover new risk loci for ECC.

**Supplementary Information:**

The online version contains supplementary material available at 10.1186/s12903-021-01623-y.

## Background

Early childhood caries (ECC) is a widespread, socio-behavioural and dental condition seen among children throughout the world [1]. Recently at the International Association of Paediatric Dentistry declaration at Bangkok 2019, ECC was defined as the presence of one or more decayed (non-cavitated or cavitated), missing or filled (due to caries) surfaces, in any primary tooth of a child under six years of age [2]. ECC is a common and mostly untreated tooth decay in preschool children with profound impact on a childs life [1]. Children with untreated ECC may suffer from pain, acute and chronic conditions including bacteraemia, early loss of primary tooth, malocclusion in the permanent dentition, low self- esteem, and failure to thrive [3, 4]. ECC continues to be one of the most prevalent chronic conditions in children especially minorities and socioeconomically disadvantaged who also have poor access to dental facilities [5,7]. The combination of the high disease burden of ECC and poor access to dental care has a great social and economic impact which affects the quality and outcomes of child oral health [4]. The prevalence of childhood dental caries in Nigerian children has been reported to be of epidemic proportion [8], ranging between 11.2% and 48.0% across the country [8]. Of great concern is the high level of untreated caries in children which is alarming with values above 80% in all parts of Nigeria. Despite decreases in the prevalence of caries among other age groups, ECC rates have remained high and for this reason it is important to determine possible explanations for the persistence of early-onset form of childhood dental caries, looking beyond established behavioural, environmental, and societal risk factors, and consider biological ones.

ECC is a disease with multiple causative factors. Some of the well documented factors implicated in the development of ECC include high levels of cariogenic micro-organisms such as *Streptococcus mutans* (which is usually transmitted to the childs oral cavity by parents or caregivers), host factors (susceptible tooth surface), salivary factors (reduced salivary flow and compromised buffering potential) and fermentable carbohydrate diet [9, 10]. Other risk indicators associated with ECC include oral hygiene practices, frequent between-meal snacks, parental attitudes, educational status of mother, socioeconomic status, chronic illnesses or special health care needs, breastfeeding and bottle-feeding habits especially at night, and frequent use of sweetened medication [2, 5,10]. In addition to environmental risk factors, genetic factors have been associated with the risk for caries [11,13]. Studies have shown associations between single nucleotide polymorphisms (SNPs) and ECC [14].

Numerous candidate-gene studies have since been conducted to investigate the role of genetics in caries aetiology in children [11, 15]. Shaffer et al. in their genome-wide association study, identified several genetic loci (ACTN2, MTR, EDARADD, MPPED2, and LPO) with suggestive evidence of association that could have plausible biological roles in childhood caries [11]. Meng et al. in their GWAS study in children and primary dentition reported three loci at the genes [interleukin 32 (IL32), galactokinase 2 (GALK2), and CUGBP, Elav-like family member 4 (CELF4)] associated with *S. mutans* carriage and severity of caries at significant levels [16]. Recently Ballantine et al. identified 13 genetic loci demonstrating suggestive evidence of association with ECC, of these 13 loci, the two most significant regions were observed on 4q32 and 20q22 marked by SNPs rs4690994 and rs439888, respectively [17]. A recent pilot GWAS of caries by Orlavo et al. conducted among African-Americans showed genetic heterogeneity. They concluded that there may be differences in the contributions of genetic variants to caries across racial groups [18].

Behavioural and environmental risk factors for ECC have been well documented [5,7, 19,21]. However, presently there are no reports on the genetic factors related to ECC in African populations. Knowledge of the genetics and genomics of ECC will help to carry out individual risk assessment, innate susceptibility and behavioural modification in order to optimize the implementation of personalized prevention and treatment [21]. Therefore, the study was designed to assess the risk factors for ECC among a cohort of Sub-Saharan African children and to investigate the genetic risk factors by genotyping single nucleotide polymorphisms that were previously reported for childhood caries. The study hypothesis was that certain gene polymorphisms increase the risk for ECC.

## Methods

### Study population and study design

This study was conducted among preschool children below 6years of age in Lagos, Nigeria, from March 2018 to October 2019. Details of study design has been reported in previous study [22]. Briefly, the study was conducted in five Local Government Areas (LGAs) of Lagos State (Mushin, Surulere, Lagos Mainland, Ikorodu and Epe). Lagos State is in the South-Western geopolitical zone of Nigeria with an estimated population of 17 million and it is the economic centre of the country. It shares boundaries with Ogun State both in the North and East and on the West by Benin Republic. In the South, it stretches for 180km along the coast of the Atlantic Ocean and occupies an area of 3577 km^2^, 22% or 787 km^2^ which consists of lagoons and creeks. It consists of 20 LGAs.

### Ethics consideration

Ethical approval for the study was obtained from the Health Research Ethics Committee of the Lagos University Teaching Hospital(LUTH), Lagos, Nigeria (ADM/DCST/HREC/APP/1669). The study was conducted according to the guidelines laid down in the Declaration of Helsinki for all procedures involving human subjects/patients. Written informed consent was obtained from the parents or legal guardian of the study participants.

### Inclusion and exclusion criteria

Children younger than 6years of age whose parents agreed to their children partaking in the research and who provided informed consent were included in the study. Children with physical or mental incapacity, those with disorders that made routine oral hygiene measures difficult, and hence are more predisposed to dental caries, those with any developmental dental anomaly and those with ongoing dental treatment were excluded.

### Sampling procedure

A multistage probability sampling technique was adopted for this study [22]. At the first stage, five LGAs (Mushin, Surulere, Lagos Mainland, Ikorodu and Epe) were selected out of the 20 LGAs in Lagos state, using simple random sampling. In the second stage, one public and one private school was selected by the simple random sampling method from each LGA. The lists of government registered public and private nursery and primary schools [23, 24] was obtained from the Lagos State Universal Basic Education Board (SUBEB) and it served as the sampling frame. Systematic random sampling method was used to select infants, toddlers and children in the nursery and primary sections of the schools, who were eligible by set criteria for the study, with the nominal roll of pupils in the selected schools serving as the sampling frame. A consent letter was sent to the parents of the children in the schools explaining the aim, scope, and importance of the study and asking for parental approval. A proforma with the study questionnaire was attached to the consent form. The consenting parents completed the questionnaire and signed the consent forms for their children to be examined and to have salivary samples taken. Children without signed parental consent forms or whose parents expressed objection to participate were excluded from the study.

### Questionnaire

The interviewer-administered questionnaire was adapted from previously validated questionnaires [5,7]. It consisted of five main parts. The first part included demographic information (childs age, gender, tribe, mothers education, fathers occupation). The second part assessed behavioural characteristics such as childs feeding patterns while the third part assessed the participants utilization of dental services. The fourth part included childs oral hygiene practices and the fifth part of the questionnaire assessed nature of childs diet and snacking habits (Additional file 1).

### Clinical examination

Detailed enrolment procedures, inclusion and exclusion criteria are reported in a previous study [7]. In brief, dental caries diagnosis was done using the World Health Organization (WHO) diagnostic criteria. The participants were examined sitting, under natural light, using sterile dental mirrors and probes by trained dentists who were calibrated. Caries was assessed by means of visual/tactile examination without radiographs. Intra examiner and extra examiner were assessed by examining 10 children with dental caries on two separate occasions with two weeks interval. The result was coded and entered into the computer. The data were then subjected to a Cohens kappa scores analysis, to determine the intra-examiner and inter-examiner variability. The intra-examiner variability scores ranged from 0.80 to 0.85 while the inter-examiner variability scores ranged from 0.81 to 0.84 for the dmft indices. Children under 6years of age, having one or more decayed (non-cavitated or cavitated), missing (due to caries), or filled tooth surfaces (dmfs) in any primary tooth were considered to have ECC. A child younger than three years of age, with one or more cavitated, missing (due to caries), or filled smooth surfaces in primary maxillary anterior teethand from ages three through five, a decayed, missing, or filled score of greater than or equal to four (age 3), greater than or equal to five (age 4), or greater than or equal to six (age 5) was considered to have SECC. Dental caries was recorded using decay, missing, filled/teeth (dmft) and surface (dmfs) index for each participating child. Children with caries were allocated into cases while children without caries were allocated into the control group. After the screening procedure, children with untreated caries were referred to the Department of Child Dental Health, LUTH for treatment and follow-up.

### Genetic analysis

Saliva samples were collected from 338 children with early childhood caries and 353 children without caries. Saliva samples were collected using Oragene-DNA Collection Kits (http://www.dnagenotek.com), labeled and shipped to the laboratory at the University of Iowa for extraction, processing and analyses. DNA was extracted from samples using Qiagen DNA Extraction Kits and sample concentration was quantified using Qubit Assays and the Qubit 2.0 Fluorometer (http://www.invitrogen.com/site/us/en/home/brands/Product-Brand/Qubit.html). XY genotyping was also used as a quality control measure to confirm that sample sex matched clinical records from collection. SNP assays for the 14 SNPs (included in a 24 assay panel required for fluidigm) of interest were designed based on human genome assembly GRCh38/hg38, 2013 and purchased through ABi/Life Technologies (www.lifetechnologies.com).

All DNA samples were diluted to an identical concentration of 1ng/ul, and underwent preamplification processes as per Fluidigm manufacturer recommendations due to the nanoscale volume of Fluidigm Integrated Fluidic Circuits (IFCs). Following preamplification, the samples were diluted to decrease reaction component concentration, to prevent reaction inhibition which may affect quality of results in subsequent steps. Additional reagents are then added in preparation for Fluidigm. In this study, we used the 192.24 Dynamic Array IFC Fluidigm chip, which allowed for 4,608 simultaneous reactions using 192 samples and 24 assays (14 of these assays are relevant and were used for the current study). Samples were randomly assigned in labeled 96-well plate maps. Each Fluidigm chip had at least two No Template Controls (NTCs), which consisted of TE Buffer only. NTCs were added to ensure the presence of negative control conditions for comparison, and to establish a point of origin in scatter plot analyses later in the study.

Prior to loading samples, the chip was primed with control line fluid, helping to maintain pressure and facilitate the closing of chip valves. Samples were then pipetted into the sample inlets and assays were placed into detector inlets. Once all samples had been introduced to the chip and all air bubbles had been eliminated, the chip was placed onto the IFC Controller RX machine for loading and mixing. The machine then applied pressure, pushing the sample and assay fluid into their respective fluid lines. Using a carry-over slug design, sample mixing was carried out as partial volumes of the first assay and sample were pushed into and combined in isolated reaction chambers. The load-mixing process occurred on the first machine over the span of 45min, after which the chip, with all samples amply mixed with their corresponding assays, underwent PCR on the FC1 Cycler in order to induce isolated reactions.

Once both machine cycles had been completed, a fluorescent image was taken of the endpoint state of the genotyping chip using the BioMark System for Genetic Analysis. The fluorescent signals were obtained through FAM and VIC channels, with FAM having an excitation peak of 495nm and an emission peak 520nm, and VIC having an excitation peak of 538nm and an emission peak of 554nm. The final image with fluorescent signals in all 4,608 reaction chambers was then plugged into Fluidigm SNP Genotyping Analysis software to generate genotype calls. Fluorescent signal brightness levels in both FAM and VIC channels were calculated and plotted on a scatter plot in the software based on relative intensity: Wang et al. [25]. With FAM on the X-axis and VIC on Y-axis, the fluorescent signals fell into four different genotype groups, homozygous FAM, homozygous VIC, heterozygous brightness between the two, and no signal negative controls.

### Data analysis

The data was entered and analyzed using IBM Statistical Package for the Social Sciences (SPSS) Version 22.0 (IBM Corp, Armonk, NY). Bivariate comparisons (t Test, ANOVA, and Chi-square tests) and Multivariate logistic regression analyses were conducted to assess associations between ECC and predictor variables. Casecontrol analyses of the genotype data was done using the additive model in PLINK. Nominal significance was set at p<0.05 and Bonferroni corrected p value was p<0.004 i.e. 0.05 divided by 14 (number of SNPs and tests, where every SNP is a test and 0.05 is significant for just one SNP/ test).

## Results

Table 1 shows the distribution of early childhood caries and severe early childhood caries among subjects. For caries free children, females made up 50.1% of the study participants, while the highest proportion of them were aged 2435months (30.3%); had mothers with>12years of formal education (64.2%) and fathers in professional/ managerial occupations (89.4%). Majority of the study participants did not receive night-time bottle feeding (92.4%); were breastfed for<12months (95.5%); had no previous dental visit (61.2%) and did not consume sugar- sweetened drinks/snacks daily (87.5). Of the 338 children with ECC, 274 (81.07%) had more than one carious tooth while 64 (18.93%) had S-ECC.

**Table 1:** Risk factors, risk indicators and socio-demographic parameters associated with early childhood caries among study subject with caries

Variables	ECC-free (n=353)	ECC (n=338)	S-ECC (n=64)	dtSD	dmftSD	p value
	N (%)					
Sex						
Male	176 (49.9)	171 (50.6)	34 (53.1)	2.792.18	2.892.17	0.191
Female	177 (50.1)	167 (49.4)	30 (46.9)	3.132.56	3.272.64	
Age (months)						
011	13 (3.7)	2 (0.6)	2 (3.1)	2.000.00	2.000.00	0.009*
1223	45 (12.7)	19 (5.6)	5 (7.8)	2.051.43	2.161.34	
2435	107 (30.3)	54 (16.0)	13 (20.3)	2.572.10	2.772.48	
3647	90 (25.5)	85 (25.1)	19 (29.7)	3.242.88	3.242.88	
4859	97 (27.5)	165 (48.8)	24 (37.5)	3.042.29	3.152.26	
6071	1 (0.3)	13 (3.9)	1 (1.6)	2.332.52	4.001.73	
Maternal education						
12years of formal education	126 (35.8)	158 (46.7)	11 (17.2)	3.212.55	3.252.52	0.577
>12years of formal education	227 (64.2)	180 (53.3)	53 (82.8)	2.872.25	2.982.23	
Paternal occupation						
Skilled/unskilled labour	37 (10.6)	159 (47.1)	29 (45.3)	2.912.44	2.972.42	0.817
Professional/managerial	316 (89.4)	177 (52.9)	35 (54.7)	2.852.02	2.992.01	
Night-time bottle feeding						
Yes	27 (7.6)	66 (19.5)	43 (67.2)	3.082.20	3.151.96	<0.001*
No	326 (92.4)	272 (80.5)	21 (32.8)	2.792.24	2.912.24	
Night-time breastfeeding						
12months	18 (5.0)	175 (51.8)	53 (82.8)	3.012.89	3.132.36	0.007*
<12months	335 (95.0)	163 (42.2)	11 (17.2)	2.191.22	2.191.22	
Previous dental visit						
Yes	137 (38.8)	80 (23.7)	25 (39.1)	2.792.11	2.822.09	0.032*
No	216 (61.2)	258 (72.3)	39 (60.9)	3.112.59	3.442.53	
Daily consumption of sugar-sweetened drinks/snacks						
Yes	44 (12.5)	151 (44.7)	53 (82.8)	3.932.51	4.002.48	0.020*
No	309 (87.5)	187 (55.3)	11 (17.2)	2.862.35	2.992.40	
Total (each subsection)		338 (100)	64 (100)	2.962.38	3.082.42	

*Significant values for dmftSD

Children aged 4859months comprised the highest proportion of subjects with ECC (165; 48.8%) and S-ECC (24; 37.5%) while those aged 3647months had significantly higher mean dt (3.242.88) while those aged 6071months had higher dmft (4.001.73).values than subjects in other age groups. Similarly, male children constituted a higher proportion of the patients with ECC (171; 50.6%) and S-ECC (34; 53.1%) while female subjects had higher dt values (3.132.56) and dmft values (3.272.64) though the association was not significant. Furthermore, those that were bottle-fed at night with formula/sugar-sweetened drinks (67.2%), those who had night-time breastfeeding12months (82.8%), those with no history of previous dental visit (60.9%) and those who daily consumed sugar-sweetened drinks/snacks (82.8%) had a higher proportion of S-ECC. They also had significantly higher mean dmft values. Similarly, children whose fathers had a professional/ managerial level occupation (54.7%) also constituted a higher proportion of the patients with S-ECC even though the association was not significant.

In the logistic regression analysis of significant predictors in the bivariate model for significant predictors of ECC, those breastfed at night12months (OR 3.30; CI 0.39, 4.75), those with no previous dental visit (OR:1.71; CI 0.24, 2.77), who used sweetened pacifiers (OR 1.85; CI 0.91, 3.79) and those who daily consumed sugar-sweetened drinks/snacks (OR 1.35; CI 0.09, 18.51) had significantly higher odds of having S-ECCTable 2.

**Table 2:** Results of the logistic regression model for ECC

	OR	CI	p
Night-time breastfeeding12months	3.30	0.39, 4.75	0.043*
No previous dental visit	1.71	0.24, 2.77	0.045*
Use of sweetened pacifiers	1.85	0.91, 3.79	0.040*
Daily consumption of sugar-sweetened drinks/snacks	1.35	0.09, 18.51	0.032*
Age	0.62	0.31, 2.03	0.053
Sex	1.01	0.67, 1.38	0.122
Paternal occupation	1.79	0.94, 3.42	0.990
Maternal education	1.11	0.34, 3.63	0.539

*Significant

**Fig. 1:**
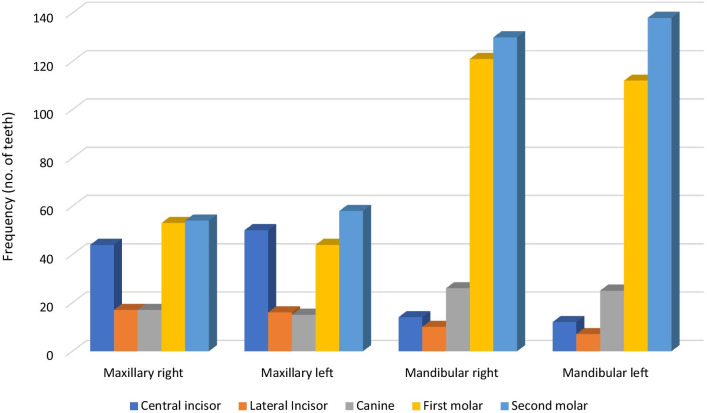
Pattern of tooth decay in children with ECC

Figure1 shows the Pattern of tooth decay in children with ECC. Primary molars accounted for the highest proportion of decayed teeth with mandibular left 2nd molars being the highest number (n=138). This was followed by the mandibular right 2nd molar (n=128) and then the mandibular right first molar (n=118). Mandibular left lateral incisors were the least carious teeth (n=6).

Table 3 shows the results of the association analyses with ECC for 14 SNPs. Two SNPs (rs11239282 and rs131777)showed significant association at the nominal significant level. (p=0.05).

**Table 3:** Single nucleotide polymorphisms associated with ECC

Current study									Ballantine study			
CHR	SNP	MAF (cases)	MAF (controls)	Minor allele	p	OR	L95	U95	OR	L95	U95	Minor allele
1	rs6702652	0.1632	0.1756	G	0.5384	0.92	0.69	1.21	0.26	0.48	0.14	G
4	rs3796983	0.3279	0.3414	G	0.5962	0.94	0.75	1.18	0.26	0.14	0.48	G
4	rs4690994	0.4347	0.4545	C	0.4591	0.92	0.75	1.14	3.5	2.09	5.95	C
5	rs1500131	0.1944	0.187	C	0.7267	1.05	0.80	1.37	3.53	2.09	5.95	C
9	rs1624327	0.405	0.3881	T	0.5201	1.07	0.86	1.33	2.91	1.79	4.73	T
10	rs932986	0.4718	0.4972	A	0.3461	0.90	0.73	1.18	2.83	1.76	4.55	A
10	rs11239282*	0.3531	0.3011	G	**0.0397**	1.27	1.01	1.59	2.91	1.8	4.73	G
11	rs12365282	0.2991	0.3324	A	0.1845	0.86	0.68	1.08	0.32	0.19	0.54	A
12	rs17104851	0.2887	0.2991	A	0.6707	0.95	0.75	1.20	5.54	2.53	12.1	A
12	rs7134614	0.2374	0.2131	C	0.2798	1.15	0.89	1.48	5.18	2.44	11	C
21	rs439888	0.2463	0.262	T	0.5019	0.92	0.72	1.17	3.6	2.1	6.2	T
22	rs7286697	0.4659	0.4618	C	0.8781	1.02	0.82	1.26	2.99	1.81	4.96	C
22	rs6000748	0.2959	0.3168	C	0.3998	0.91	0.72	1.14	3.87	2.09	7.19	C
22	rs131777*	0.4241	0.4801	C	**0.0373**	0.80	0.65	0.99	3.33	1.95	5.66	C

MAF=Minor allele frequency * and Bold indicates SNPS with p<0.05

## Discussion

Dental caries is the most prevalent disease in children, and it is a serious public health problem, especially in socially disadvantaged populations. Certain socio-demographic, psycho-social, microbiological and behavioural risk factors contribute to the development of carious lesions. The identification of groups at risk for ECC is critical for designing preventive initiatives and initiating early treatment.

It was observed in this study that ECC was associated with prolonged night-time breastfeeding. This is similar to what was reported in an Australian study by Haag et al. [26]. In the study, they observed an association between exclusive breastfeeding for six months and continued breastfeeding for 24months with 1.45 higher caries prevalence. Recently, there have been several systematic reviews with conflicting reports about the effect of breastfeeding on ECC. A recent systematic review by *Moynihan* et al. [27] concluded that breastfeeding until two years of age did not increase caries risk. In another review, Tham et al. [28] reported that breastfeeding protected against ECC but that breastfeeding beyond 12months of age increased the risk of ECC, this is also in agreement with the report of the meta-analysis by Cui et al. [29], where they concluded that breastfeeding greater than 12months of age is associated with higher risk of ECC. Although links have been associated with breastfeeding beyond age one year and ECC, it is important to consider other confounding factors such as night-time feeding, use of fluoride, utilization of dental services and other related factors. The difficulty in linking association of ECC directly with prolonged breastfeeding due to confounding factors has led to recommendation by specialists and societies to encourage breastfeeding, provided that teeth are cleaned, consumption of sugar is reduced and infants are not allowed to fall asleep with the breast [30].

Breastfeeding has many well-established benefits for both mother and baby, thus, potential effects of breastfeeding on caries should be mitigated by improving oral hygiene and access to oral healthcare [26].

There was a higher prevalence of ECC and S-ECC in children who daily consumed sugar-sweetened drinks and snacks and children who were bottle-fed with formula/sweetened drinks at night. These findings corroborated previous similar studies carried out among Nigerian children [5,8]. A recent study reported that despite the lower consumption of sugar among children from low-income countries, the prevalence of ECC was high, this could possibly be explained by the impact of the frequency of consumption when compared to the quantity of consumption of sugar [31]. Also, there is access to cheaper high sugar containing meals in low-resourced settings which is a readily accessible alternative diet [32]. Daily and frequent consumption of low quantity of sugar can increase the risk of ECC and therefore it is important to control intake of sugar from early childhood to prevent unwanted dietary habits which tend to remain in adulthood. The risk factors for ECC are multifactorial as observed in our study, the aetiological mechanisms that we observed may however additionally involve gene-environment interactions.

Furthermore, targeted genotyping of 14 suggestive SNPs on a cohort of 338 children with ECC and 353 children without caries was performed and replicated the signals for two SNPs (rs11239282 and rs131777). Although, the effect size for the rs11239282 in the current study is lower than what was reported by Ballantine, the effect sizes are in the same direction and suggestive of an increased risk for ECC at this locus across populations. Opposite effect sizes for rs131777 were observed. In the present study cohort, the effect size suggests a reduced risk while in the Ballantine study, they reported an increased risk at this locus for the same allele. This may be due to chance if the associations are spurious or due to different environmental contexts altering the impact of this variant between the two studies if the associations are indeed real. For the 12 SNPs that were not replicated, differences in effect sizes and direction between the two populations were observed. The lack of replication for the remaining 12 SNPs could indicate that the previously observed associations were due to chance, or could be evidence of possible differences that exist for the genetic architecture of ECC across populations. These difference may be in the minor allele frequency and the haplotype blocks which is smaller in Africans compared to Europeans. The latter explanation is supported by the fact that population specific signals for complex traits that have been widely reported [33,35].

The rs11239282 is an eQTL for the Ras association domain family member gene (*RASSF4*) and differential expressions have been reported in tissues such as brain, heart, breast, colon, stomach and minor salivary glands. The *RASSF4* gene is involved in the promotion of apoptosis, cell cycle arrest and signal transduction. It also functions as a potential tumour suppressor. RASSF4 was shown to be expressed in the dental pulp in a microarray study [36]. Expression in relevant dental tissue supports its role in tooth development and caries.

The rs131777 is an eQTL for the choline kinase beta gene (*CHKB*) with differential expression of the gene in tissues like muscles, whole blood, fibroblast and the lungs. *CHKB* is involved with phospholipid metabolism and regulates catalysation of the first step of phosphatidylethanolamine and phosphatidylcholine biosynthesis [37, 38]. Mutations in this gene have been reported in individuals with muscular dystrophy [39, 40]. It is uncertain how CHKB is involved in ECC and possible that it is not the gene of interest and perhaps another gene in the same topologically associated domain as the rs131777 is involved.

The aetiological relevance of specific microbiome groups appears to vary between different populations [41], suggesting that genetic risk for oral microbiome might also vary between populations. The composition of the humanoral microbiomeis influenced by the hostgeneticbackground while shared and non-shared environmental factors are significant drivers oforalmicrobial communities, especially, in connection tooraldisease andmicrobiomematuration [42]. Additional studies to explore these possibilities are required as we plan studies on personalized care and prevention.

A limitation of this study was that ethnicityspecific results were not examined for sub-analysis, while we also did not determine the exposure of the children to topical or systemic sources of fluoride.

## Conclusion

A high prevalence of S-ECC (18.93%) and a gradient of distribution of ECC along low socioeconomic status, poor utilization of dental services and unhealthy nutritional practices were observed. The study also showed evidence of hereditability of ECC among African population with replication of two previously reported SNPs for ECC. Larger samples sizes are needed to confirm the results of the genetic analysis and to conduct genome wide studies in order to discover new risk loci for ECC.

## Supplementary Information

**Additional file 1**.Questionnaire.

## Data Availability

The data supporting the findings of this manuscript will be made available by the corresponding author, without undue reservation, to any qualified researcher.

## Abbreviations

dft: Number of decayed or filled primary teeth
dmft: Number of decayed, missing, or filled primary teeth
ECC: Early childhood caries
GWAS: Genome-wide association study
MAF: Minor allele frequency
SNP: Single nucleotide polymorphism
S-ECC: Severe early childhood caries
WHO: World health organization

## References

[1] Hallett KB, ORourke PK (2003). Social and behavioural determinants of early childhood caries. Aust Dent J.

[2] Pitts N, Baez R, Diaz-Guallory C (2019). Early childhood caries: IAPD Bangkok Declaration. Int J Paediatr Dent.

[3] Wagner Y, Knaup I, Knaup TJ (2020). influence of a programme for prevention of early childhood caries on early orthodontic treatment needs. Clin oral Invest.

[4] Olatosi OO, Sote EO (2012). Causes and pattern of tooth loss in children and adolescents at the Lagos University Teaching Hospital. Nig Q J Hosp Med.

[5] Onyejaka N, Amobi E (2016). Risk factors of early childhood caries among children in Enugu, Nigeria. Braz Res Paediatr Dent Integr Clin.

[6] Folayan MO, Kolawole KA, Oziegbe EO, Oyedele T, Oshomoji OV, Chukwumah NM (2015). Prevalence, and early childhood caries risk indicators in preschool children in suburban Nigeria. BMC Oral Health.

[7] Olatosi OO, Inem V, Sofola OO, Prakash P, Sote EO (2015). The prevalence of early childhood caries and its associated risk factors among preschool children referred to a tertiary care institution. Niger J Clin Pract.

[8] Folayan MO, Chukwumah NM, Onyejaka N, Adeniyi AA, Olatosi OO (2014). Appraisal of the national response to the caries epidemic in children in Nigeria. BMC Oral Health.

[9] Tinanoff N, Baez RJ, Diaz Guillory C, Donly KJ, Feldens CA, McGrath C (2019). Early childhood caries epidemiology, aetiology, risk assessment, societal burden, management, education and policy: global perspective. Int J Paediatr Dent.

[10] Vadiakas G (2008). Case definition, aetiology and risk assessment of early childhood caries (ECC): a revisited review. Eur Arch Paediatr Dent.

[11] Shaffer JR, Wang X, Feingold E (2011). Genome-wide association scan for childhood caries implicates novel genes. J Dent Res.

[12] Wang X, Shaffer JR, Zeng Z, Begum F, Vieira AR, Noel J, Anjomshoaa I, Cuenco KT, Lee MK (2012). Genome-wide association scan of dental caries in the permanent dentition. BMC Oral Health.

[13] Shaffer JR, Feingold E, Wang X, Lee M, Tcuenco K, Weeks DE, Weyant RJ, Crout R, McNeil DW, Marazita ML (2013). GWAS of dental caries patterns in the permanent dentition. J Dent Res.

[14] Piekoszewska-Zieztek P, Turska-Szybka A, Olczak-Kowalczyk D (2017). Single nucleotide polymorphism in the aetiology of caries: systematic literature review. Caries Res.

[15] Divaris K (2017). Precision dentistry in early childhood: the central role of genomics. Dent Clin North Am.

[16] Meng Y, Wu T, Billings R, Kopycka-Kedzierawski DT, Xiao J (2019). Human genes influence the interaction between Streptococcus mutans and host caries susceptibility: a genome-wide association study in children with primary dentition. Int J Oral Sci.

[17] Ballantine JL (2018). Exploring the genomic basis of early childhood caries: a pilot study. Int J Paediatr Dent.

[18] Orlova E, Carlson JC, Lee MK, Feingold E, McNeil DW, Crout RJ (2019). Pilot GWAS of caries in African-Americans shows genetic heterogeneity. BMC Oral Health.

[19] Folayan M, Sowole A, Kola-Jebutu A (2007). Risk factors for caries in children from South Western Nigeria. J Clin Pediatr Dent.

[20] Abiola AA, Eyitope OO, Sonny OJ, Oyinkan OS (2009). Dental caries occurrence and associated oral hygiene practices among rural and urban Nigerian pre-school children. J Dent Oral Hyg.

[21] Olatosi OO, Sote EO (2014). Association of early childhood caries with breastfeeding and bottle feeding in Southwestern Nigerian children of preschool age. J West Afr Coll Surg.

[22] Olatosi OO, Oyapero A, Onyejaka NK (2020). Disparities in caries experience and socio-behavioural risk indicators among private school children in Lagos. Nigeria. Pesqui Bras Odontopediatria Cln Integr.

[23] Lagos State Government Basic Statistical Hotline. from:http://mepb.lagosstate.gov.ng/wp-content/uploads/sites/29/2017/01/Basic-Stat-Hotline-Y2010.pdf. Accessed 11 Jan 2019.

[24] List of Primary and Secondary Schools in Lagos State|Nigeria Top List [Internet]. https://nigeriatoplist.com/list-of-primary-and-secondary-schools-in-lagos-state/. Accessed 2 Dec 2018

[25] Wang J, Lin M, Crenshaw A (2009). High-throughput single nucleotide polymorphism genotyping using nanofluidic dynamic arrays. BMC Genomics.

[26] Haag DG, Jamieson LM, Hedges J, Smithers LG (2019). Is there an association between breastfeeding and dental caries among three-year-old Australian aboriginal children?. Nutrients.

[27] Moynihan P, Tanner LM, Holmes RD, Hillier-Brown F, Mashayekhi A, Kelly SAM, Craig D (2019). Systematic review of evidence pertaining to factors that modify risk of early childhood caries. JDR Clin Trans Res.

[28] Tham R, Bowatte G, Dharmage SC, Tan DJ, Lau MX, Dai X, Allen KJ, Lodge CJ (2015). Breastfeeding and the risk of dental caries: a systematic review and meta-analysis. Acta Paediatr.

[29] Cui L, Li X, Tian Y, Bao J, Wang L, Xu D, Zhao B, Li W (2017). Breastfeeding and early childhood caries: a meta-analysis of observational studies. Asia Pac J Clin Nutr.

[30] Branger B, Camelot F, Droz D, Houbiers B, Marchalot A, Bruel H (2019). Breastfeeding and early childhood caries. Review of the literature, recommendations, and prevention. Arch Pediatr.

[31] Folayan MO, El Tantawi M, Ramos-Gomez F, Sabbah W (2020). Early childhood caries and its associations with sugar consumption, overweight and exclusive breastfeeding in low, middle and high-income countries: an ecological study. Peer J.

[32] Mobley C, Marshall TA, Milgrom P, Coldwell SE (2009). The contribution of dietary factors to dental caries and disparities in caries. Acad Pediatr.

[33] Beaty TH, Murray JC, Marazita ML, Munger RG, Ruczinski I, Hetmanski JB (2010). A genome wide association study of cleft lip with / without cleft palate using case-parent trios of European and Asian ancestry identifies MAFB and ABCA4 as novel candidate genes. Nat Genet.

[34] Butali A, Mossey PA, Adeyemo WL, Eshete MA, Gowans LJJ, Busch TD (2019). (2019) Genomic analyses in African populations identify novel risk loci for cleft palate. Hum Mol Genetics.

[35] Adeyemo AA, Zaghloul NA, Chen G (2019). *ZRANB3* is an African-specific type 2 diabetes locus associated with beta-cell mass and insulin response. Nat Commun.

[36] Torun D, Torun Z, Demirkaya K, Sarper M, Eli MP, Avcu F (2017). Microarray analysis of the gene expression profile in triethylene glycol dimethacrylate-treated human dental pulp cells. Niger J Clin Pract.

[37] Gallego-Ortega D, Ramirez de Molina A, Ramos MA, Valdes-Mora F, Barderas MG, Sarmentero-Estrada J, Lacal JC (2009). Differential role of human choline kinase alpha and beta enzymes in lipid metabolism: implications in cancer onset and treatment. PLoS ONE.

[38] Mitsuhashi S, Ohkuma A, Talim B, Karahashi M, Koumura T, Aoyama C, Kurihara M, Quinlivan R, Sewry C, Mitsuhashi H, Goto K, Koksal B, Kale G, Ikeda K, Taguchi R, Noguchi S, Hayashi YK, Nonaka I, Nishino I (2011). A congenital muscular dystrophy with mitochondrial structural abnormalities caused by defective de novo phosphatidylcholine biosynthesis. Am J Hum Genet.

[39] Castro-Gago M, Dacruz-Alvarez D, Pintos-Martnez E, Beiras-Iglesias A, Arenas J, Martn M, Martnez-Azorn F (2016). Congenital neurogenic muscular atrophy in megaconial myopathy due to a mutation in CHKB gene. Brain Dev.

[40] Oliveira J, Negro L, Fineza I, Taipa R, Melo-Pires M, Fortuna AM, Gonalves AR, Froufe H, Egas C, Santos R, Sousa MJ (2015). New splicing mutation in the choline kinase beta (CHKB) gene causing a muscular dystrophy detected by whole-exome sequencing. Hum Genet.

[41] Johansson I, Witkowska E, Kaveh B, Holgerson PL, Tanner ACR (2016). The microbiome in populations with a low and high prevalence of caries. J Dent Res.

[42] Gomez A, Espinoza JL, Harkins DM (2017). Host genetic control of the oral microbiome in health and disease. Cell Host Microbe.

