# Supplementary material for: Replication of GWAS significant loci in a sub-Saharan African Cohort with early childhood caries: a pilot study
Source: BMC Oral Health. 2021 May 20;21:274. doi: 10.1186/s12903-021-01623-y (PMC8139096; doi:10.1186/s12903-021-01623-y)
Supplement: Supplementary file 1 — Additional file 1.Questionnaire. [file 12903_2021_1623_MOESM1_ESM.pdf]

Appendix A: Questionnaire

Socio-demographics

1. Study ID .... Date…………..
2. Current age (years) .... Date of birth __D ____M _______Year
3. Gender [ ] Male [ ] Female
4. Telephone number ________________________________
5. State of origin/Local Government Area _____________________/______________
6. Tribe [ ] Yoruba [ ] Ibo [ ] Hausa [ ] Fulani [ ] Efik [ ] Tiv [ ] other
7. Family history of dental caries [ ] Yes [ ] No
8. Mother's level of education (or stepmother, guardian or any other female adult living with you)
   i. No formal schooling [ ]
   ii. Primary school [ ]
   iii. Secondary school [ ]
   iv. College/university [ ]
   v. No female adult in household [ ]
9. Fathers' occupation (or stepfather, guardian or any other male adult living with you)
   i. Professional [ ]
   ii. Managerial [ ]
   iii. Skilled, non-manual [ ]
   iv. Skilled manual [ ]
   v. Unskilled [ ]
   vi. No male adult in household [ ]

Infant feeding pattern

10. What method of feeding did you use for your child? [ ] breastmilk only [ ] bottle-feeding only [ ] breast and bottle feeding
11. If you breastfed your child, how long did you breastfeed? [ ] <3 months [ ] 3-6 months [ ] 7-12 months [ ] >12 months
12. Did you breastfeed your baby at will/on demand? [ ] yes [ ]no
13. Did you breastfeed your baby at night? [ ] yes [ ] no
14. At what age did you wean your child from bottle-feeding? [ ] <6 months [ ] 6 – 12months [ ] > 12 months
15. Did you put your baby to sleep with bottle containing milk formular/juice or any sugar drink in the mouth? [ ] yes [ ] no

Utilization of dental services

16. Have you ever taken your child to the dentist (tooth doctor) for examination or has a dentist ever attended to your child? [ ] yes [ ] no
17. If your answer is' no' what is the reason? **(you can pick more than one answer)**
    i. I don't know there are dentists (tooth doctor) [ ]
    ii. I don't have any tooth problem [ ]
    iii. I don't have the money to go to the dentist [ ]
    iv. There is no dental facility near my location [ ]
    v. I use traditional alternatives [ ]
    vi. I don't have the time [ ]
    vii. Others-----------------------------
18. If you have taken your child to the dentist, what was the reason?
    i. Pain or trouble with tooth, gums or mouth [ ]
    ii. For treatment [ ]
    iii. Routine check [ ]
    iv. I don't remember [ ]
    v. Others ----------------------

Oral hygiene practices

19. How often do you clean or does your child clean his/her teeth?
    i. Never [ ]
    ii. Several times a month [ ]
    iii. Once a week [ ]
    iv. Several times a week [ ]
    v. Once daily [ ]
    vi. 2 or more times daily [ ]

20. which of the following does your child use to clean his/her teeth and gum **(more than one answer is allowed)**

	Yes	no
Toothbrush		
Chewing stick		
Toothpick		
Charcoal		
Ground glass		
Floss		
Others		

21. Do you use toothpaste to clean your child's teeth? [ ] Yes [ ] No
22. If yes, does the toothpaste contain fluoride? [ ] Yes [ ] No [ ] I don't know

Consumption of Sugar snacks and drinks

23. How often do you eat or drink any of the following foods, even in small quantities?

	Never	Several times a month	Once a week	Several times a week	Everyday	Several times a day
Fresh fruit						
Biscuit, cakes, puff-puff, meatpie, gala						
Coke, fanta, caprisone, bobo, ribenna,						
Sweets, chewing gum,						
Milk with sugar						
Pap, custard, cereal with sugar						

24. Did your child use a pacifier? [ ] yes [ ]no
25. If yes, did you coat it with [ ] honey [ ] sugar [ ] others-------------
26. Did you give your child or is your child currently on any vitamin supplement? [ ] yes [ ] no
